# Self-Harm and Interpersonal Violence-Related Injuries: Retrospective Analysis of the American College of Surgeons Trauma Quality Programs Data

**DOI:** 10.5811/westjem.42044

**Published:** 2025-08-29

**Authors:** Ayman El-Menyar, Ahammed Mekkodathil, Rafael Consunji, Sandro Rizoli, Tarik S Abulkhair, Ruben Peralta, Rifat Latifi, Hassan Al-Thani

**Affiliations:** * Hamad Medical Corporation, Department of Surgery, Clinical Research, Trauma and Vascular Surgery, Doha, Qatar; † Weill Cornell Medical College, Department of Clinical Medicine, Doha, Qatar; ‡ Hamad Medical Corporation, Department of Surgery, Trauma Surgery, Injury Prevention Program, Doha, Qatar; § Hamad Medical Corporation, Department of Surgery, Trauma Surgery, Doha, Qatar; || Universidad Nacional Pedro Henriquez Urena, Department of Surgery, Santo Domingo, Dominican Republic; # College of Surgery, Department of Surgery, Pristina, Kosovo

## Abstract

**Introduction:**

Violence-related injuries (VRI) such as interpersonal violence, intimate-partner violence, and self-harm injuries present a significant public health challenge in the United States. We aimed to explore interpersonal-violence and self-harm injuries, focusing on demographic disparities (age and sex) and mechanisms of injury, including firearm-related violence.

**Methods:**

We conducted a retrospective study of VRIs among the US civilian population between 2017–2021. Data were extracted from the American College of Surgeons (ACS) Trauma Quality Programs Participant Use Files. We identified VRIs using the International Classification of Diseases, 10th Rev, with Australian modification E-code series. The dataset was categorized and compared by age, sex, ethnicity, violence intent, and mechanism of injury. This study was a secondary data analysis reporting interpersonal-violence and self-harm injuries among trauma cases from the national trauma database.

**Results:**

The total number of trauma patients in the ACS database was 5,483,016 between 2017–2021 (1.1 million/year). The final analysis included 584,417 (11%) patients with VRIs (interpersonal violence and self-harm), with a mean age of 35 years; 82% were male, 45% White, and 42% Black. Interpersonal violence accounted for 88% of injuries, while 12% were self-harm, with firearm-related violence the most common mechanism of injury (35%). Firearm-related interpersonal violence was common among younger individuals (19–39 years), while non-weaponized interpersonal violence was prevalent among older individuals (≥ 60 years). Blacks had a higher rate of firearm-related interpersonal violence (51%), and Whites had a greater frequency of non-weaponized interpersonal violence. There were 43,089 deaths (7.4%), with 68% resulting from interpersonal-violence and 32% from self-harm injuries. Firearm-related injuries (interpersonal violence and self-harm combined) accounted for 78% of all VRI-related deaths. Mortality was higher in males (7.7%) than in females (5.9%) (P < .001).

**Conclusion:**

There is a significant burden of violence-rated injuries in the US, particularly affecting males, racial minorities, and vulnerable age groups. Firearm-related injuries are the leading cause of death in both interpersonal-violence and self-harm cases. The increase in VRIs during the COVID-19 pandemic highlights the urgent need for targeted public health interventions focused on firearm safety, violence prevention, and mental health support.

## INTRODUCTION

Violence-related injuries (VRI) remain a significant public health concern; it is collectively responsible for 4.4 million deaths (8% of all deaths) worldwide.^1^ Self-harm or self-inflicted injury is the main mechanism of VRIs leading to death, followed by interpersonal violence.^1^ In the 15–29 years age group, interpersonal violence is the third leading cause of death, while self-harm ranked fourth.^1^ In addition to the immediate consequences, VRIs have a lifelong impact on individuals and communities, including mental health challenges, disabilities, healthcare costs, and diminished individual productivity.^2^,^3^

In the United States, violence-related fatalities remain a major concern, with homicide and suicide accounting for 14 and 7.8 deaths per 100,000 individuals, respectively, in 2020.^4^ Beyond mortality, nonfatal VRIs are also significant. Approximately 1.6 million individuals experience non-fatal interpersonal-violence injuries annually.^5^ The economic burden of violence is considerable, with the combined costs of homicides and suicides estimated at $670 billion^6^ and the inflation-adjusted medical and work-loss costs for VRIs among adults reaching $49.5 billion in a single year.^7^ Additionally, about 1.4 million adults sought emergency department (ED) care for VRIs, representing 1.6% of all adult ED visits in the US. Visits to the ED were disproportionately higher among young adults 18–25 years of age, males, non-Whites, uninsured or publicly insured patients, and residents of high-poverty urban areas.^7^

The demographic pattern of VRIs in the US aligns with global trends, as worldwide statistics reveal that males accounted for 84% of VRI fatalities between 2010–2015.^8^ In contrast, females represented 16% of VRI deaths, equivalent to an annual average of 64,000 deaths.^8^ However, analysis of ED visits for interpersonal-violence injuries shows only a slight male predominance, with rates of 4.9 vs 4.2 visits per 1,000 individuals for males and females, respectively.^9^ The highest rate of interpersonal violence-related ED visits was observed among young adults aged 18–24 years (9.2 visits per 1,000 individuals annually), declining slightly in the 25–44 age group (7.7 visits per 1,000) and further among older adults.^9^ Until 2015, most fatalities from interpersonal violence were attributed to firearm-related injuries.^10^ By 2022, firearm-related injuries accounted for over 48,000 deaths, approximately 132 deaths per day, with more than half due to suicides and over 40% resulting from firearm homicides.^11^

While VRIs, which include both interpersonal violence and self-harm, remain major public health concerns, significant knowledge gaps persist regarding the demographic disparities, mechanism of injury, and evolving trends influenced by the COVID-19 pandemic. In particular, the prominent role of firearm-related injuries in both interpersonal violence and self-harm contexts requires focused attention to guide prevention efforts. In this study we aimed to explore the patterns of interpersonal violence and self-harm injuries in the US, focusing on demographic disparities, firearm-related mechanisms, and pandemic-associated trends. We sought to provide a comprehensive understanding to inform targeted public health interventions, violence prevention strategies, and firearm injury-prevention efforts.

**Population Health Research Capsule**

What do we already know about this issue?

*Violence-related injuries (VRI) such as interpersonal violence and self-harm have significant mortality and economic burden, with firearms a major factor in the US.*

What was the research question?

*What were the demographic disparities, mechanisms, and trends of interpersonal-violence and self-harm injuries in the US from 2017–2021?*

What was the major finding of the study?

*Among VRIs (88% interpersonal and 12% self-harm), firearms represented 35% of mechanism of injury. Males had higher VRI mortality than females (7.7 vs 5.9%), P < .001.*

How does this improve population health?

*Identifying high-risk groups will guide targeted interventions for violence prevention, firearm safety, and mental health support to reduce morbidity and mortality.*

## METHODS

We conducted a retrospective study of VRIs in the US between 2017–2021. Data pertaining to VRIs within the civilian population were extracted from the American College of Surgeons (ACS) Trauma Quality Programs Participant Use Files. All data were obtained from an existing database (secondary data analysis); no individual chart abstraction was performed; therefore, specific chart-review methodologies, such as those described by Worster and Bledsoe, were not applicable.^12^ Over 99.5% of the data pertains to patients within the US; a small minority of international patients also contribute to the dataset. However, due to data protection regulations, specific geographic information on patients or facilities was not available. Since this study involved secondary analysis of de-identified data provided by the ACS files, institutional review board approval was not required.

The selected external cause codes (E-codes) from the *International Classification of Diseases, 10^th^ Rev*, with Australian modification (ICD-10-AO) in the trauma registry—specifically T43, T51–T65, T71, T74, T76, X71–X83, X92–X99, Y00–Y08, and Y35–Y38—were used to identify VRIs. We excluded from the study injuries sustained by military personnel during military operations, war operations, and other legal interventions. We also excluded cases lacking age and sex data (only males and females were included) and those with missing information regarding the mechanism of injuries. This study reported intentional injuries focusing on VRIs (both interpersonal violence and self-harm) from all trauma cases in the database. The data were categorized based on violence intent, type of trauma, and mechanism of injury. The intent of trauma were interpersonal violence and self-harm. The violence intent, type of trauma, and mechanism of injury were assigned based on ICD-10-AO E-codes. Trauma types included blunt, penetrating, burn, and other forms (eg, asphyxiation, drowning, poisoning). Mechanism of injury included blunt assaults (eg, motor vehicle collisions, falls), penetrating injuries (eg, firearms, sharp objects), burns, and other mechanisms.

The World Health Organization defines violence as the intentional use of physical force or power, either threatened or actual, resulting in injury, death, psychological harm, mal-development, or deprivation.^13^ This inclusive definition spans interpersonal, self-directed, and collective violence. Interpersonal violence involves the intentional use of force against others by an individual or small group, manifesting physical, sexual, or psychological harm. Family or partner violence encompasses child maltreatment, dating and intimate partner violence, and elder maltreatment. Community violence involving non-family individuals includes youth violence, bullying, assault, and institutional violence in settings like schools and workplaces.^13^

In our study, non-weaponized interpersonal violence refers to physical harm inflicted through bodily force without the use of external objects such as firearms, blunt instruments, or sharp weapons. This category includes acts of physical abuse, sexual abuse, and physical assault involving personal force, such as punching, kicking, slapping, or other forms of direct contact. A perpetrator of maltreatment and neglect is an individual responsible for causing harm or failing to provide adequate care to a vulnerable person, such as a child, elderly individual, or dependent adult. Perpetrators are often caregivers or family members who abuse their responsibilities, leading to physical, emotional, or developmental harm to the victim.

### Statistical Analysis

We used descriptive statistics to summarize the demographic profiles, mechanism of injury, and mortality rates of VRIs in different age groups, ethnicities, and sex. Continuous variables, such as age, were summarized using means and standard deviations, while categorical variables, including sex, race, mechanism of injury, and mortality, were reported as frequencies and percentages. We employed chi-square tests to assess the association between categorical variables, such as sex, race, and mechanism of injury, with interpersonal violence and self-harm. Continuous variables such as age were compared using *t*-tests. The age groups were 0–18, 19–39, 40–60, and > 60. We compared injuries among US ethnicities (White, Black, Asian, Hispanic/Latino, and others). We employed the Python 3.0 pandas library (Python Software Foundation, Wilmington, DE) for data compilation and performed statistical analysis using SPSS v21 (IBM Corp, Armonk, NY). Case fatality rate was calculated as the proportion of deaths among patients with a specific injury mechanism, expressed as a percentage.

## RESULTS

**Figure 1:**
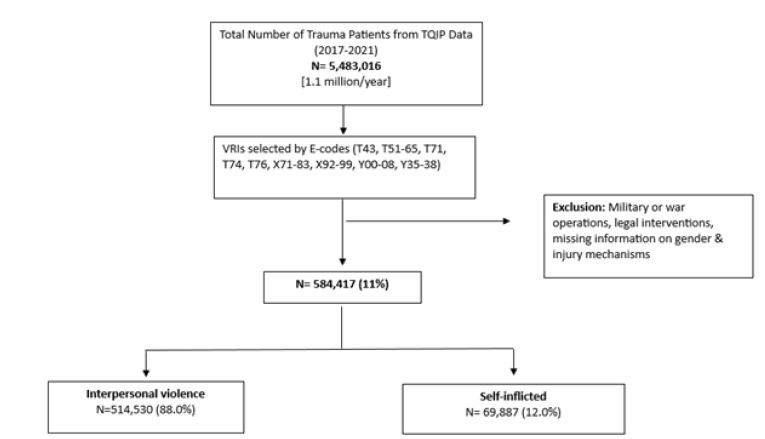
Selection flowchart of violence-related injury cases from the dataset of the American College of Surgeons Trauma Quality Programs Participant Use File, 2017–2021. *TQIP*, Trauma Quality Improvement Program; *E-code*, external cause code

Between 2017–2021, the total number of trauma patients in the ACS database was 5,483,016 (1.1 million/year). The final analysis included 584,417 (11%) patients with VRIs (Figure 1).

**Figure 2:**
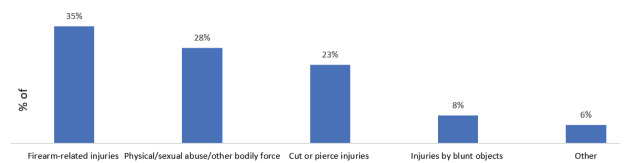
Distribution of violence-related injury mechanisms in the United States (2017–2021).

The mean age of patients was 35 years (±15), and 82% were males (Table 1). The racial distribution was 45% White and 42% Black. Interpersonal violence accounted for 88% of VRIs, while self-harm constituted 12%. Firearm-related injuries were the most common mechanism of injury, comprising 35% of all VRIs, followed by non-weaponized interpersonal violence (28%) and cut or pierce injuries (23%) (Figure 2).

Mortality among patients with VRIs was 7.4%, with a significantly higher case fatality rate observed among males (7.7%) compared to females (5.9%) (*P* < .001). Firearm-related injuries accounted for 78% of total VRI mortality (80% in males vs 66% in females, *P* = .001). Firearm-related interpersonal violence mortality was 52% of all VRI mortality (55% in males vs 40% in females, *P* = .001). In contrast, firearm-related self-harm mortality accounted for 25% of all VRI mortality (25% in males vs 26% in females) of total mortality (55% in males vs 40% in females). In contrast, firearm-related self-harm mortality of all VRI mortality was similar, 25% in males and 26% in females (Table 2).

The age-stratified analysis demonstrated that firearm-related interpersonal violence was most prevalent among children 0–18 years of age (51%) and younger adults (19–39 years of age; 42%), whereas non-weaponized interpersonal violence was more common in older adults ≥ 60 years of age (53%) (Table 3). Firearm-related self-harm injuries were also more common among older adults (46%) when compared to other age groups (*P* = .001). Similarly, racial disparities were evident. Blacks had disproportionately higher rates of firearm-related interpersonal violence, while Whites had a higher frequency of non-weaponized interpersonal violence. In self-harm, firearm injuries were more common in Whites, whereas cut or pierce injuries were more common among Asians (Table 4).

Trends over time revealed an increase in the proportion of VRIs during the pandemic years, rising from 18.9% in 2017 to 21.8% in 2020 before slightly declining to 21.4% in 2021. Mortality associated with VRIs also increased from 18.5% in 2017 to 23.2% by 2021 (Table 5).

## DISCUSSION

This study provides a comprehensive analysis of VRIs in the US, revealing significant demographic disparities and highlighting the dominant role of firearm-related injuries in both interpersonal violence and self-harm. Young males, particularly Black individuals, were disproportionately affected by firearm-related interpersonal violence, while non-weaponized interpersonal violence was more common among older adults and White individuals. These findings emphasize the need for targeted interventions addressing firearm violence and demographic vulnerabilities.

In 2011, approximately 1.4 million people worldwide lost their lives due to violence, with 35% of these deaths attributed to interpersonal violence.^14^ Sumner et al^5^ described the burden of interpersonal violence in the US based on data from multiple health and law enforcement surveillance systems. Homicide rates significantly decreased from a peak of 10.7 per 100,000 in 1980 to 5.1 in 2013. Non-fatal interpersonal violence rates dropped by 45% from 1992 to 2012. Partner violence rates show that 32% of women and 28% of men have experienced physical violence in their lifetime. Intimate partner violence rates against women have decreased by 72% since 1994. Elder abuse affects 11% of community-dwelling adults ≥ 60 years of age.^5^

The US Centers for Disease Control and Prevention WISQARS compilation (Web-based Injury Statistics Query and Reporting System) shows the top causes of death for individuals 1–44 years of age in the US from 1981–2022. In 1981, unintentional injury was the leading cause with 58,500 deaths, homicide was third with 17,900 deaths, and suicide was fifth with 15,900 deaths. By 2012, suicide became the second leading cause with 18,200 deaths, while homicide dropped to fifth with 12,300 deaths. In 2020, homicide rose to third with 18,800 deaths, and suicide remained second with 22,400 deaths. In 2021, COVID-19 became the third leading cause with 23,700 deaths, but suicide stayed second with 23,900 deaths. By 2022, suicide remained second with 23,400 deaths, and homicide was third with 18,600 deaths.^15^ Our study demonstrates that upward trends in VRI rates and mortality highlight a growing burden of VRIs across both sexes, with males consistently representing a larger share of the affected population. From 2020, VRIs and mortality demonstrated a rise in the post-COVID-19 years. Injuries increased from 19.1% in 2019 to 21.8% in 2020 and remained high at 21.4% in 2021. Mortality rose from 17.9% in 2019 to 23.2% by 2021, reflecting the impact of the pandemic on violence trends.

Previous reports show that older adults > 60 years of age accounted for 2% of ED visits following intimate partner violence, more frequently males and Black, sustained injuries due to cutting, lacerations, and injuries to the upper extremity.^16^,^17^ Meyer et al^18^ also reported that the older adult victims of firearm-related violence were predominantly Black (50%) and males (85%). Hiranniramol et al^19^ studied patients presenting with VRIs and found that the average age was 33 years, with 70–80% being Black males. Homicide rates varied significantly by age and sex, with males 15–29 years of age experiencing nearly five times the homicide rate of females.^20^ Our study also demonstrated that young males are more likely to experience violence-related deaths, particularly from firearms. Interestingly, global data show that female homicide rates doubled between ages 5–14 and 15–29 and in women ≥ 70 years of age. However, our study demonstrated high mortality rates among both older adults (12%) and children < 19 years of age (80%). Gitto et al^21^ reported that the most common cause of death among female victims was firearm-related wounds, followed by sharp force traumas, asphyxia, and blunt force injuries.

Carmichael et al^10^ studied more than 18,000 homicides based on data from the National Violent Death Reporting System. They found that most of the deaths were due to firearms (72%) or sharp objects (13%). More than half (53%) of the patients who died on the scene were neither seen by emergency medical services nor transported to an ED, most probably due to the severity of the injury or remaining unnoticed for a long time by someone other than the suspect. Seven of ten medically treated firearm injuries in youths result from interpersonal violence, with males 14–24 years of age accounting for 88% of these cases.^22^

Dowd et al^23^ reported that 18% of patients with penetrating firearm-related self-harm injuries had major psychiatric illnesses, 8% had alcohol use disorder, 6% had drug use disorder, and a few (0.6%) had dementia. The study reported an upward trend in the proportion of patients with major psychiatric illnesses, from 16% in 2013 to 19% in 2016, with a peak in 2015 at 21%. A high mortality rate (50%) was observed in firearm-related self-harm injuries when compared with attempted murder (7%) and accidental discharge of guns (3%). Blacks were more likely to get injured due to attempted murder than Whites (85% vs 40%), whereas rates of accidental discharge of gunshot (47% vs 13%) and self-harm injuries were higher in White patients.^23^

Young and Xiang^24^ reported that Blacks were heavily impacted by firearm homicide, with homicide age-adjusted death rates almost seven times higher when compared to Whites. A scoping review of 19 studies conducted by Marineau et al^25^ indicated that Black men in the US face disproportionate risks of firearm-related interpersonal violence, primarily due to structural and social inequities. The review identified key risk factors across the social-ecological framework, such as firearm possession at the individual level, gang membership at the relationship level, socioeconomic status at the community level, and historical racist policies at the societal level.

Tennakoon et al^26^ studied domestic and intimate partner violence in the US and found that young males were more likely to become victims. Our study demonstrated that male mortality rates were significantly higher. Firearm-related VRIs remain a significant mechanism in both interpersonal violence and self-harm cases, contributing to 78% of total deaths. The gun homicide rate in the US was nearly 25 times higher than in other high-income countries.^27^ Bell et al^28^ reported a stable overall intentional injury rate among adolescents but highlighted an increase in self-harm injuries, particularly among younger females. Our study showed a high proportion of firearm-related self-harm deaths among older adults.

Hoefer et al^29^ studied 41,239 pediatric firearm-trauma patients and observed a significant rise in firearm-related self-harm incidence over a 12-year period, with a substantial proportion being White (67%), teenagers (90%), and males (87%). The firearm-related self-harm mortality rate was reported as 44%, with head gunshot wounds and higher Injury Severity Score significantly linked to mortality.^29^ DiVietro et al^30^ reported that non-Hispanic White children died at a rate that was 1.3 times greater than expected based on their proportion in the general population and were 2.6 times more likely to die by firearm suicide than non-Hispanic Black children.

The COVID-19 pandemic further exacerbated the firearm violence crisis. Shannon et al^31^ observed a rise in firearm-related violence during the pandemic despite an overall 10% decrease in assault-related hospitalizations. Stevens et al^32^ found that pediatric firearm injuries surged during the pandemic, with a cumulative increase in 2020 compared to historical data. Risinger et al^31^ reported that from 2011–2021, Jefferson County, Kentucky, recorded 6,043 firearm injuries. During the COVID-19 period, 4,574 years of potential life were lost due to the pandemic, compared to 9,722 years lost to all-cause gun violence. In the pre-COVID-19 period, there were 5,723 years of potential life lost due to all-cause gun violence.^33^ Firearm violence incidents, injuries, and deaths surged following society’s re-emergence from the COVID-19 pandemic.

Additionally, mass shootings increased despite the initial decline during the pandemic, indicating that the “reopening phenomenon” exacerbated an already significant national firearm epidemic.^34^ This study period from 2017–2021 includes pre-pandemic, pandemic, and post-pandemic eras, providing the pattern of VRIs across these distinct timeframes. During the pandemic era (2020–2021), VRIs demonstrated a notable increase compared to pre-pandemic years. In 2020, the overall proportion of VRIs peaked, and this trend was consistent across sexes. Mortality associated with VRIs also demonstrated an increase during the pandemic years. These findings indicate that the pandemic era coincided with increased VRIs and mortality, possibly influenced by factors such as social isolation, economic challenges, increased stress, and disruptions to healthcare and social support systems.

Also of concern is the high rate of trauma recidivism, wherein patients suffer subsequent VRIs. Studies have reported five-year recidivism rates ranging from 25–44%, with one study reporting that recidivists accounted for 16% of trauma visits.^35^,^36^ El-Menyar et al^37^ analyzed 9,855 trauma patients and found that 8% had a history of violence prior to admission at a New York hospital. These patients were more likely to be younger, male, Black, Hispanic, and covered by low-income primary insurance payors compared to non-assault trauma patients. Multivariate logistic regression indicated that predictors of violence included being Black and male, with low-income primary insurance payor status, and Asian ethnicity, drug use, alcohol intoxication, and smoking.

Factors such as the mechanism of injury and behaviors are closely linked to higher recidivism rates.^38^, ^39^ This predisposition to repeat VRIs creates a worsening cycle of trauma, particularly with penetrating injuries and firearm-related incidents, which significantly increase the risk of death with each subsequent injury.^35^,^38^ These findings highlight the need for targeted public health interventions focused on firearm violence prevention among high-risk populations, particularly young males and racial minorities disproportionately affected by interpersonal violence. In addition, mental health support programs aimed at reducing self-harm injuries, especially among older adults, are essential. Interventions should focus on promoting firearm safety, preventing violent conflicts, and supporting community-based programs to reduce the burden of VRIs. Public health efforts must also consider the demographic disparities and the impact of the COVID-19 pandemic on violence trends.

Schwartz et al^40^ examined firearm-related injuries in the US civilian population using prehospital data from the National Emergency Medical Services Information Systems database. Their findings revealed that prehospital responders most frequently classified injuries to four anatomical regions as Critical-Red: 63% of chest injuries; 54% of abdominal injuries; 48% of neck injuries; and 42% of back injuries. In our study, the head was the most frequently injured body region, accounting for 49% of injuries. Neck injuries were reported in 11% of cases, while chest injuries comprised 28%. Injuries to the abdomen, pelvis, or spine were noted in 25% of cases.

A recent review by Sakran and Lunardi^41^ highlights the critical role that surgeons play, emphasizing hemorrhage control, expeditious transport to a trauma center, and even prehospital blood administration as examples of systems coordination between EMS and trauma centers that can prevent deaths from VRIs. They also cite the need to fully understand the social and political determinants of firearm injury to decrease the number of VRIs. Hospital-based, violence prevention programs are of utmost value as studies showed that exposure to firearm violence increases the likelihood by twofold that a young person will be engaged in violence within two years with a higher retaliatory injury risk (88 times).^42^

## LIMITATIONS

The retrospective design relies on existing data, which may be subject to reporting biases or missing information. The study lacks information on socioeconomic factors, mental health status, and other potential risk factors that could inform more comprehensive prevention strategies. Future research should incorporate these variables and explore the intersectionality of various risk factors to develop more holistic and effective interventions. The lack of specific geographic information is a notable constraint and limits our understanding of geographical patterns and disparities in VRIs across different areas. Moreover, this study only reports trauma activations. Therefore, it likely has an underestimation of the total scope of interpersonal and self-harm violence-related injuries, much of which does not rise to the level of trauma activation at designated hospitals. In addition, individuals who died before hospital arrival may not be fully captured in the dataset, further contributing to the underestimation of the overall burden of violence-related injuries. Future research should incorporate more detailed geographic data, socioeconomic factors, mental health status, and other risk determinants to enable a more comprehensive analysis and targeted prevention strategies.

## CONCLUSION

These findings highlight the increasing burden of violence-related injuries in the US, especially among males, racial minorities, and vulnerable age groups. Firearm-related injuries remain the leading cause of death in both interpersonal violence and self-harm cases. The recent rise in VRIs during the pandemic period emphasizes the need for focused public health interventions addressing firearm safety, violence prevention, and mental health support.

## Figures and Tables

**Table 1:** Description of patients with violence-related injuries in the United States (2017–2021) (N = 584,417)

Variable	Values
Mean age	35 ± 15 years
Sex	
Male	477,781 (81.8%)
Female	106,636 (18.2%)
Race	
White	253,521 (45.1%)
Black	236,538 (42.0%)
Asian	6,944 (1.2%)
Hispanic/Latino	43,870 (7.8%)
Other	21,820 (3.9%)
Intent of violence	
Interpersonal violence	512,834 (87.8%)
Self-inflicted/self-harm	71,583 (12.2%)
Mechanism of injury	
Firearm-related injuries	202,121 (34.6%)
Physical/sexual abuse/other bodily force	165,128 (28.3%)
Cut or pierce injuries	136,252 (23.3%)
Injuries by blunt objects	48,234 (8.3%)
Other	17,098 (2.9%)
Injured body region	
Head	245,654 (49.0%)
Neck	52,633 (10.5%)
Chest	139,271 (27.8%)
Abdomen, pelvis or spine	127,411 (25.4%)
Upper limb	157,395 (31.4%)
Lower limb	124,957 (24.9%)
Transport mode	
Ground ambulance	437,552 (75.3%)
Helicopter ambulance	39,188 (6.7%)
Fixed-wing ambulance	1,937 (0.3%)
Private/public vehicle	88,519 (15.2%)
Police	9,884 (1.7%)
Other	4,136 (0.7%)
Work-related injuries	5,261 (0.9%)
Prehospital cardiac arrest	22,212 (3.8%)
Alcohol screening	354,430 (61.3%)
Mean GCS in hospital	13.4± 3.7
Median hospital length of stay	3 (1–1)
Death in emergency department	9,769 (1.7%)
Total mortality	43,089 (7.4%)

*GCS*, Glasgow Coma Scale

**Table 2:** Distribution of violence-related injuries by intent, mechanism, sex, and associated mortality (N = 584,417).

Intent of Violence-related Injury (VRI)	Mechanism of VRI	Cases (% of total cases)	Male, N (%)	Female, N (%)	P-value (prevalence)	Deaths, N (%)	Male mortality, N (%)	Female mortality, N (%)	P-value (mortality)
Interpersonal Violence (N = 512,834; 87.8%)	Firearm injuries	180,457 (30.9%)	157,437 (33.0%)	23,020 (21.6%)	.001	22,564 (52.4%)	20,065 (54.5%)	2,499 (39.9%)	.001
	Non-weaponized	165,128 (28.3%)	127,026 (26.6%)	38,102 (35.7%)	.001	2,540 (5.9%)	1,933 (5.2%)	607 (9.7%)	.001
	Cut/pierce injuries	106,363 (18.2%)	91,895 (19.2%)	14,468 (13.6%)	.001	2,696 (6.3%)	2,258 (6.1%)	438 (7.0%)	.001
	Blunt injuries	47,396 (8.1%)	40,713 (8.5%)	6,683 (6.3%)	.001	517 (1.2%)	443 (1.2%)	74 (1.2%)	.001
	Perpetrator of maltreatment and neglect	6,412 (1.1%)	3,098 (0.6%)	3,314 (3.1%)	.001	310 (0.7%)	176 (0.5%)	134 (2.1%)	.001
	Burn injuries	794 (0.1%)	509 (0.1%)	285 (0.3%)	.001	18 (0.04%)	11 (0.03%)	7 (0.1%)	.001
	Other IPV	6,284 (1.1%)	3,961 (0.8%)	2,323 (2.2%)	.001	164 (0.4%)	99 (0.3%)	65 (1.0%)	.001
Self-harm VRI (N = 71,583; 12.2%)	Firearm injuries	21,664 (3.7%)	17,915 (3.7%)	3,749 (3.5%)	.001	10,920 (25.3%)	9,305 (25.3%)	1,615 (25.8%)	.001
	Cut/pierce injuries	29,889 (5.1%)	21,387 (4.5%)	8,502 (8.0%)	.001	631 (1.5%)	522 (1.4%)	109 (1.7%)	.001
	Jumping from height	9,172 (1.6%)	6,019 (1.3%)	3,153 (3.0%)	.001	1,097 (2.5%)	814 (2.2%)	283 (4.5%)	.001
	Asphyxiation	1,711 (0.3%)	1,289 (0.3%)	422 (0.4%)	.001	649 (1.5%)	479 (1.3%)	170 (2.7%)	.001
	Burn injuries	956 (0.2%)	631 (0.1%)	325 (0.3%)	.001	130 (0.3%)	99 (0.3%)	31 (0.5%)	.001
	Other SH	8,191 (1.4%)	5,901 (1.2%)	2,290 (2.1%)	.001	853 (2.0%)	620 (1.7%)	233 (3.7%)	.001
Total		584,417 (100%)	477,781 (100%)	106,636 (100%)		43,089 (100%)	36,824 (100%)	6,265 (100%)	

**Table 3:** Age-stratified distribution of violence-related injuries in patient population in the United States (2017–2021).

Type of violence-related injuries	Mechanism of injuries	Age 0–18 years (N = 50,151)	19–39 years (N = 289,492)	40–59 years (N = 127,608)	Age ≥ 60 years (N = 31,369)	P-value
Interpersonal-violence-related injuries (IPV)	Firearm-related injuries	25,632 (51.1%)	122,286 (42.2%)	28,004 (21.9%)	3,828 (12.2%)	.001
	Non-weaponized assaults	14,152 (28.9%)	72,776 (25.1%)	49,150 (38.5%)	16,257 (52.7%)	
	Cut or pierce injuries	5,934 (11.8%)	65,993 (22.8%)	29,146 (22.8%)	5,024 (16%)	
	Injuries by blunt objects	1,621 (3.2%)	22,666 (7.8%)	18,031 (14.1%)	4,938 (15.7%)	
	Perpetrator of maltreatment and neglect	1,307 (2.6%)	1,615 (0.6%)	931 (0.7%)	430 (1.4%)	
	Other mechanisms	1,145 (2.3%)	4,156 (1.4%)	2,346 (1.8%)	622 (2.0%)	
Self-inflicted/Self-harm injuries (SH)		Age 0–18y (N= 5,611)	19–39y (N= 34,978)	40–59y (N = 19,321)	Age ≥ 60y (N = 9.574)	
	Firearm-related injuries	1,846 (32.9%)	9,512 (27.2%)	5,682 (29.4%)	4,425 (46.2%)	.001
	Cut or pierce injuries	1,654 (29.5%)	15,244 (43.6%)	9,060 (46.9%)	3,791 (39.6%)	
	Jumping from height	1,103 (19.7%)	5,123 (14.6%)	2,240 (11.6%)	668 (7.0%)	
	Injuries by blunt objects	80 (1.4%)	489 (1.4%)	213 (1.1%)	54 (0.6%)	
	Other mechanisms	928 (16.5%)	4,610 (13.2%)	2,126 (11.0%)	636 (6.6%)	

**Table 4:** Race-stratified distribution of violence-related injuries in patient population in the United States (2017–2021).

Type of violence-related injuries	Mechanism of injuries	White (N= 203,461)	Black (N = 227,533)	Asian (N = 5,408)	Hispanic/Latino (N = 40,227)	Other (N = 17,177)	P-value
Interpersonal-violence related injuries (IPV)	Firearm-related injuries	42,039 (20.7%)	115,466 (50.7%)	1,100 (20.3%)	11,795 (29.3%)	5,015 (29.2%)	.001
	Non-weaponized assaults	89,368 (43.9%)	47,506 (20.9%)	2,190 (40.5%)	13,013 (32.3%)	5,903 (34.4%)	
	Cut or pierce injuries	41,310 (20.3%)	44,462 (19.5%)	1,228 (22.7%)	10,245 (25.5%)	4,090 (23.8%)	
	Injuries by blunt objects	22,636 (11.1%)	15,731 (6.9%)	664 (12.3%)	4,090 (10.2%)	1,658 (9.7%)	
	Perpetrator of maltreatment and neglect	3,448 (1.7%)	1,812 (0.8%)	80 (1.5%)	428 (1.1%)	247 (1.4%)	
	Others	4,660 (2.3%)	2,556 (1.1%)	146 (2.7%)	656 (1.6%)	264 (1.5%)	
		White (N = 50,060)	Black (N = 9,005)	Asian (N = 1,536)	Hispanic/Latino (N = 3,643)	Other (N = 2,378)	
Self-harm injuries	Firearm-related injuries	16,349 (32.7%)	2,689 (29.9%)	209 (13.6%)	772 (21.2%)	580 (24.4%)	.001
	Cut or pierce injuries	21,657 (43.3%)	3,254 (36.1%)	789 (51.4%)	1,782 (48.9%)	1,022 (43.0%)	
	Jumping from height	6,756 (11.5%)	1,665 (18.5%)	336 (21.9%)	573 (15.7%)	452 (19.0%)	
	Injuries by blunt objects	549 (1.1%)	152 (1.7%)	14 (0.9%)	55 (1.5%)	31 (1.3%)	
	Other mechanisms	5,749 (11.5%)	1,245 (13.8%)	188 (12.2%)	461 (12.7%)	293 (12.3%)	

**Table 5:** Trends of violence-related injuries and mortality in the United States (2017–2021).

	Number and % percentage of violence-related injured person (total)	Number and % percentage of violence-related injured person (Females)	Number and % percentage of violence-related injured person (Males)	Mortality
2017	110,375 (18.9%)	19,333 (18.1%)	91,042 (19.1%)	7,955 (18.5%)
2018	109,621 (18.8%)	19,771 (18.5%)	89,850 (18.8%)	7,803 (18.1%)
2019	111,840 (19.1%)	20,733 (19.4%)	91,107 (19.1%)	7,718 (17.9%)
2020	127,608 (21.8%)	23,498 (22.0%)	104,110 (21.8%)	9,609 (22.3%)
2021	124,973 (21.4%)	23,301 (21.9%)	101,672 (21.3%)	10,004 (23.2%)
